# Accurate Calibration in Multi-Material 3D Bioprinting for Tissue Engineering

**DOI:** 10.3390/ma11081402

**Published:** 2018-08-10

**Authors:** Enrique Sodupe-Ortega, Andres Sanz-Garcia, Alpha Pernia-Espinoza, Carmen Escobedo-Lucea

**Affiliations:** 1 EDMANS Group, Department of Mechanical Engineering, University of La Rioja, San José de Calasanz 31, Edificio Departamental, 26004 Logroño, Spain; enrique.sodupeo@unirioja.es (E.S.-O.); alpha.pernia@unirioja.es (A.P.-E.); 2 Division of Pharmaceutical Biosciences, University of Helsinki, Viikinkaari 5 E, P.O. Box 56, 00014 Helsinki, Finland

**Keywords:** additive manufacturing, synthetic polymer, bioprinting, multi-material microextrusion, bioink

## Abstract

Most of the studies in three-dimensional (3D) bioprinting have been traditionally based on printing a single bioink. Addressing the complexity of organ and tissue engineering, however, will require combining multiple building and sacrificial biomaterials and several cells types in a single biofabrication session. This is a significant challenge, and, to tackle that, we must focus on the complex relationships between the printing parameters and the print resolution. In this paper, we study the influence of the main parameters driven multi-material 3D bioprinting and we present a method to calibrate these systems and control the print resolution accurately. Firstly, poloxamer hydrogels were extruded using a desktop 3D printer modified to incorporate four microextrusion-based bioprinting (MEBB) printheads. The printed hydrogels provided us the particular range of printing parameters (mainly printing pressure, deposition speed, and nozzle *z*-offset) to assure the correct calibration of the multi-material 3D bioprinter. Using the printheads, we demonstrated the excellent performance of the calibrated system extruding different fluorescent bioinks. Representative multi-material structures were printed in both poloxamer and cell-laden gelatin-alginate bioinks in a single session corroborating the capabilities of our system and the calibration method. Cell viability was not significantly affected by any of the changes proposed. We conclude that our proposal has enormous potential to help with advancing in the creation of complex 3D constructs and vascular networks for tissue engineering.

## 1. Introduction

**Figure 1:**
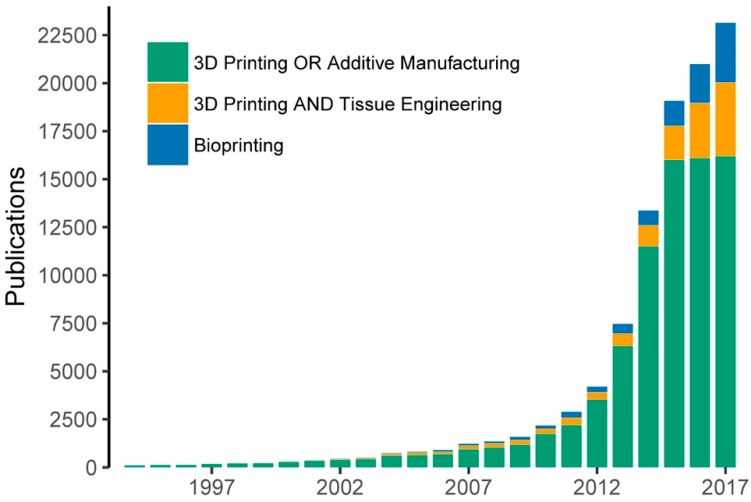
Evolution of bioprinting and 3D printing publications measured by the number of Google Scholar hits. The keywords utilized for the searching included: “3D printing” OR “additive manufacturing”, “3D printing” and “tissue engineering” and “bioprinting”.

The rise of three-dimensional (3D) printing in the last three decades has permitted the arrival of a new manufacturing technology called 3D bioprinting for organ and tissue engineering (TE) [1,2,3,4]. This technology aims to deposit multiple biomaterials, growth factors, and living cells with precise control over their compositions, spatial distribution and architecture [5]. Since the appearance of the first bioprinting studies in 2003 introduced by Wilson and Boland [6], the field has experienced a growing interest by the scientific community in the last decade. The rapid increase in the number of related publications provides evidence of this tendency (Figure 1).

Today, allograft organ transplantation is still the only therapy effective against organ failures, but relatively simple implantable tissue constructs have been printed and successfully transplanted into animal models [7]. These works bring great hope for those patients who are looking for alternatives to the organ transplantation methods. Looking forward, the challenge remains of how to reproduce the complex cellular organization and micro-environment of an entire solid organ. This is still well beyond the capabilities of currently available bioprinting technologies [8].

Three main bioprinting technologies have been developed: inkjet-, laser- and microextrusion-based bioprinting (MEBB) systems. Each of them has been utilized in several biological applications, offering different features in terms of cell viability, deposition speed, print resolution, scalability, cost or materials compatibility [9]. MEBB is the most extended technology because of its versatility and fast deposition of a wide range of bioinks, which enables the rapid generation of large-scale constructs [10,11,12]. Although excessive printing pressures could reduce cell viability, it is an excellent method for depositing high cell densities in several candidate bioinks [13]. The bioinks can be defined as formulation of biomaterials, biological molecules and cells processed using bioprinting technologies [14,15]. Most of the studies in 3D bioprinting have traditionally been limited to the use of one or two bioinks at one time, which is perhaps an oversimplification that limits the structural, material and biological potential of this technology [16].

Employing multiple building and sacrificial biomaterials and cells types in a single biofabrication session seems to be the right way of addressing the complexity of organ engineering and producing outstanding advances in the field [17,18,19]. Multi-material bioprinters have recently been developed by several research groups [7,11,12,20,21,22]. These bioprinting systems normally incorporate up to three or four printheads to perform multi-material extrusion like the open-source solution utilized by the authors in this study. To the best of our knowledge, advances in multi-material bioprinting will enable researchers to integrate intricate perfusable channels inside of complex shape constructs, and create constructs with several different cell densities, among other advantages. A more detailed study in multi-material bioprinting [8], using stem-cell-laden bioinks, alongside a network of reinforcing poly(*ε*-caprolactone) (PCL), led to the biofabrication of so-called developmentally inspired templates of bone tissue microfibers.

All of this cannot be accomplished without answering fundamental questions such as the ideal properties of the bioinks and the relationships between the bioprinting process parameters and the print resolution and fidelity [13]. In the case of MEBB, some previous research studies have correlated bioprinting parameters and printed outcomes. Wang et al. showed that optimized printing parameters such as bioink concentration, nozzle speed and extrusion rate produced poly(lactic-*co*-glycolic acid) (PLGA) scaffolds [23]. Mixtures of Gel-Alg were investigated by He and his coworkers to find the optimal values of air pressure, feed rate, and layer height to assure proper printing quality [13]. Suntornnond et al. used poloxamers to develop a mathematical model to correlate print resolution with process parameters [24]. Similarly, a prediction model was obtained by Trachtenberg et al. to determine the suitability of poly-propylene fumarate for MEBB [25] while Ting et al. examined the effect of PLGA composition and printing parameter on print resolution [26]. However, today, there is no a definite method to calibrate multi-material 3D bioprinters as well as to determine their final print resolution. Understanding how parameters such as printing speed and nozzle height affect the print resolution is vital not only for the shape of the printed constructs but also for their mechanical properties. When encapsulating cells, selecting the optimal printing parameters will reduce the adverse effect of the viscoelastic stresses on the cell viability [27,28].

In this paper, we advance in the development of the multi-material 3D bioprinting by proposing a method that analyzes the influence of the main printing parameters and accurately controls the print resolution. We anticipate that a significant increase on printing speed and quality of the constructs using the multi-material bioprinter is due to the use of an automatic calibration system. Poloxamer 407 (P407) hydrogels with different fluorescent inks were printed into different complex constructs for finding the optimal printing parameters. This allowed us to emulate the bioprinting of four materials, but, at the same time, also remove other secondary factors such as excessive swelling or temperature dependence. The proposed method was also tested printing a mixture of gelatin-alginate (Gel-Alg), a more cell-friendly bioink. Cell-laden Gel-Alg and P407-based bioinks were printed in a single session. After printing, cell viability of stem cells embedded in the Gel-Alg was analyzed to verify the effects of the calibration. The results demonstrated that our proposal has huge potential to help in creating large multi-material 3D constructs and potential vascular networks for tissue engineering.

## 2. Materials and Methods

### 2.1. Bioprinting System Incorporating Four Printheads

**Figure 2:**
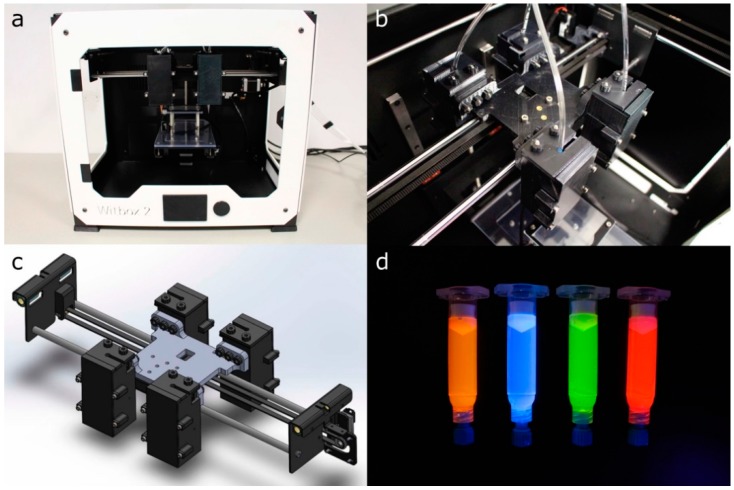
(**a**,**b**) general and detailed view of the modified Witbox 2 3D printer with four bioprinting printheads; (**c**) 3D CAD design of the 3D printer *x*-carriage; (**d**) 5 mL syringe barrels loaded with dyed P407 hydrogels.

The experiments were performed using a desktop open-source 3D printer Witbox 2 (BQ, Madrid, Spain) modified for extruding hydrogels at 24 °C (Figure 2a). The mechanical resolution of the 3D printer is up to 20 μm according to the manufacturer’s specifications. The Witbox 2 movements follow a Cartesian dimensional coordinate system, in which the printheads are moving across the *xy* horizontal plane while the printing platform only moves vertically (*z*-axis). The Witbox 2 was modified by substituting the standard fused deposition modeling nozzle in the *x*-carriage for four pneumatic-based MEBB printheads (Figure 2b,c). The four printheads’ movements are controlled using open-source Rumba electronics (Reprap Universal Mega Board with Allegro driver; RepRapDiscount, Hong Kong, China).

The printing pressure of the four printheads can be independently adjusted using individual air pressure regulators (ARP20K-N01BG-1Z; SMC, Tokyo, Japan). Hydrogel deposition in each printhead is controlled by opening and closing the solenoid valve (VT307-6DZ1-01F-Q; SMC, Tokyo, Japan) connected to the metal-oxide-semiconductor field-effect transistor (MOSFET) terminals of the Rumba controller board.

### 2.2. G-Code Generation and Printing Software

The software employed to control the modified bioprinter and the bioprinting process was comprised of several tools. First of all, a modification of Marlin firmware (v1.1) was loaded into the 3D printer main board [29]. The modified firmware allowed us to manage and coordinate all the activities of the 3D printer, including the movement of the four printheads and the deposition of the bioinks.

A computer-aided design (CAD) software (SolidWorks; Dassault Systems, v2016) was utilized to create the 3D models for bioprinting and generate the final stereolithography (STL) files. The open-source slicing software Slic3r (v1.2.9) [30] was utilized for G-code generation. Slic3r is mainly utilized in FDM and therefore it is not designed to operate pneumatic printheads. For that reason, custom post-processing Perl scripts were required to transform the original G-Code to the particular characteristics of the multi-material 3D bioprinter used. The four printheads moved according to G-code instructions, depositing biomaterials where they were initially programmed. Finally, the G-code was sent to the bioprinter using Repetier-Host (v1.6.2) software [31], which was also in charge of monitoring the bioprinting process.

### 2.3. Multi-Material Bioprinting Procedure and Calibration

**Figure 3:**
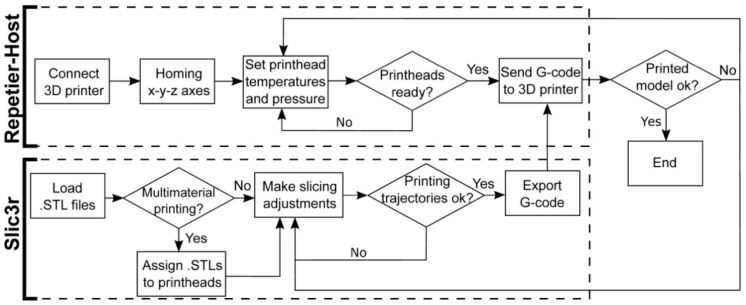
Flowchart of the procedure for the G-code generation and the interaction with the 3D bioprinter interface.

**Figure 4:**
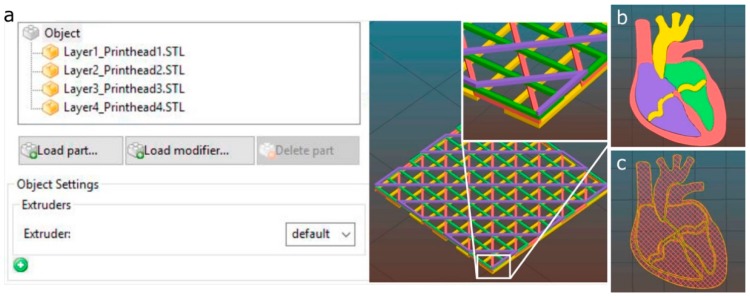
Multi-material printheads assignment using Slic3r software. (**a**) porous structure with four layers stacked, each one assigned to a different printhead; (**b**) 3D model of a heart section composed of four parts; (**c**) printhead trajectories calculated by the slicing software using a porous infill.

Figure 3 describes the procedure to prepare a 3D model for the multi-material bioprinting process. This procedure starts opening the STL files containing the original geometry with the slicing software. In case a multi-material printing process is desired, several STL files should be generated, each of them assigned to the particular printhead that will print that part of the geometry. The assigning operation is performed in Slic3r using the “Settings” button. Each STL file will be displayed in a list on the left-hand side of the window and assigned to a specific printhead (Figure 4a).

When several printheads are assigned, the 3D model visualization will appear with a different color for each printhead (Figure 4). If only one printhead is utilized, a single STL will be required. Once the printing settings are introduced (deposition speed, infill pattern, number of perimeters, etc.), the G-code is generated and sent to the 3D printer through the Repetier-Host.

**Figure 5:**
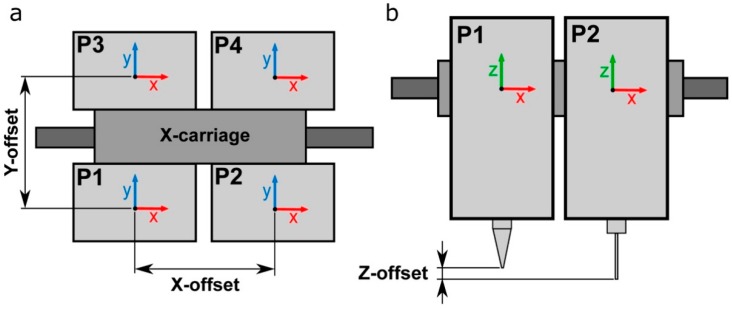
Schematic representation of the (**a**) *xy*-offsets and the (**b**) *z*-offset of four printheads P1/P4.

*xy* offsets of the 3D printer utilized were configured according to Figure 5a. When using multiple printheads, the original offset coordinates of the first printhead (P1) are set to zero (*x* = 0, *y* = 0). Then, the *xy* offset coordinates of every additional printhead must be determined with respect to the coordinates of P1. Every offset must be entered in the slicing software to compensate for the misalignments between the printheads. Depending on the particular printer used and the configuration of its printheads, the values of the offset coordinates can be very different.

*z*-offset between various printheads also represents a crucial point for multi-material calibration (Figure 5b). A *z*-homing push button was installed in the 3D multi-material bioprinter to perform the automatic calibration of the *z*-offsets. This configuration allows us to use nozzles of different types and heights. In addition, this automatic system reduces drastically the time required to start the printing process because there is no necessity to perform any manual adjustment and the whole calibration process is done at once.

### 2.4. Hydrogel Preparation

Poloxamer 407 (P407, Pluronic^®^ F127; Sigma-Aldrich, Madrid, Spain) was prepared at 40 wt % by weighing the quantity of polymer required and mixing in cold Milli-Q water at 4 °C. P407 powder was added gradually to MilliQ water to facilitate the dilution and stirred vigorously for 3 h using a magnetic stirrer at 4 °C. Once the solution was homogenized, it was centrifuged and stored overnight at 4 °C to remove air bubbles. P407 prepared solutions were always stored at 4 °C until further use.

Gelatin from porcine skin (G1890; Sigma-Aldrich) and sodium alginate from brown algae (A0682; Sigma-Aldrich) were dissolved in phosphate buffered saline (PBS) without salts at 10 wt % and 4 wt % respectively. A solution of 5%Gel-2%Alg was prepared by blending. The pH of the solution was adjusted to 7.2–7.4. Solutions were mixed using vortex and centrifuged at 1000 rpm for 1 min to remove air bubbles.

Four different fluorescent dyes (see clear differences in fluorescence under UV light at the Figure 2d) were utilized to improve the visualization of P407 and Gel-Alg (except in the case of using cells to avoid cytotoxicity) bioinks: orange (1:100; IFWB-33; Risk Reactor, Santa Ana, USA), clear blue (1:500; IFWB-C0; Risk Reactor), yellow-green (1:1000; IFWB-C8; Risk Reactor) and red (1:1000; IFWB-C7; Risk Reactor).

### 2.5. Cell Isolation and Culture

Human adipose derived mesenchymal stem cells (hASCs) were isolated from lipoaspirates of young healthy donors undergoing aesthetic surgery (from 18 to 35 years-old), following written informed consent and Research Ethical Board approval by Clinica Isabel Moreno and Hospital General Foundation, Valencia, Spain. Donors were previously screened for Human Immunodeficiency Virus (HIV), hepatitis C and other infectious diseases. hASCs were expanded following the protocol described by Escobedo-Lucea et al. [32] and harvested with Tryple^®^ (Invitrogen, Carlsbad, NM, USA) at 85% confluence.

### 2.6. Bioprinting Cell-Laden Constructs Using Gel-Alg Blends

hASCs were mixed with the bioink (cell density of 10^6^ cells/mL) by gentle pipetting to create a homogeneous suspension that was transferred into a 5 mL Luer-lock syringe (Nordson EFD, Alfafar, Spain) and closed with a piston (SmoothFlow; Nordson EFD). Extrusion was performed under controlled air pressure. The cell-laden bioinks were deposited into class slides through a 25G tapered nozzle (Nordson EFD) at a printing speed of 15 mm s^−1^. The 3D-printed constructs were finally crosslinked in 3 wt % calcium chloride (CaCl_2_; Wako, Tokyo, Japan) for 6 min and then washed three times with phosphate buffer (PBS) and replaced with growth medium, Dulbecco’s modified Eagle’s medium (DMEM, Invitrogen) supplemented with 6% human serum.

### 2.7. Cell Viability Assay

Cell viability in the printed constructs was assessed by live/dead assay (R37601; Life Technologies, Carlsbad, NM, USA) according to manufacturer’s instructions. Briefly, after printing, and crosslinking, samples were washed three times with PBS, stained with live green (A) (Calcium-AM; 0.5 μL/mL) and dead red (B) (ethidium homodimer; 2 μL/mL), and incubated for 15 min at RT. Fluorescence images of printed samples were captured 1 h and 24 h after deposition under confocal microscope (Olympus FV1200, Olympus, Tokyo, Japan). Data are representative of the printed samples of four layers stacked images.

### 2.8. Calibration Models for the Multi-Material Bioprinting Process

**Figure 6:**
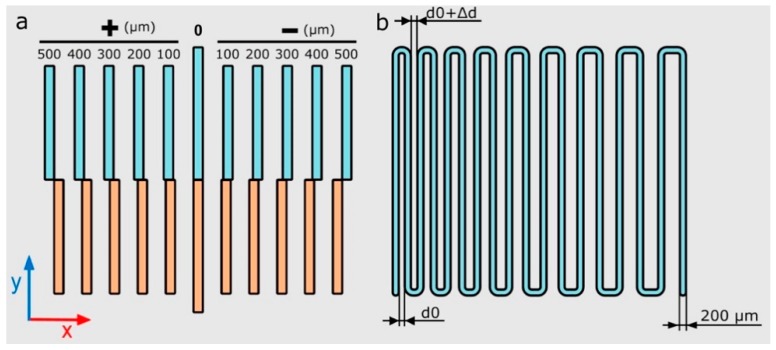
Schematic illustration of the calibration models *xy*-offset pattern (**a**) and printing pressure dependent zigzag path (**b**) where the distance *d*0 = 200 μm, and the variation Δ*d* = 20 μm.

Two main calibration models were proposed to adjust the four printheads’ *xy* positions with respect to each other and define the optimal printing pressure. These models aim to determine the printability and final print resolution in multi-material bioprinting systems. The proposed calibration 3D models were designed using the CAD modeling software SolidWorks (Dassault Systems, v2016), and exported as STL files. A detailed description and justification of the calibration models are given in the following paragraphs:

- *xy*-offset pattern (calibration model 1): straight lines were printed in the *x* and *y* directions using two different printheads (Figure 6a). *xy* offsets of the four printheads were calculated with regard to the first printhead (P1). For that reason, half of the straight lines were printed using P1 and the other half were printed using a different printhead (P2, P3 or P4).
- Zigzag path (calibration model 2): a continuous zigzag was printed using each printhead in other to determine the correct printing pressure and speed (Figure 6b). An increasing distance of 20 μm was separated between all of the lines (*Δd*) with a separation between lines ranging from 200 μm to 500 μm. The optimal printing pressure was determined when all the printed lines did not overlap and were printed forming continuous strands.

Both calibration models were printed using 40 wt % P407 on 50 × 75 × 1 mm glass slides (Corning Inc., New York, NY, USA). The P407 was loaded into 3 mL and 5 mL syringe barrels (Nordson EFD) at 4 °C and extruded at 24 °C. *xy*-offset calibration model was printed using tapered nozzles with three different inner diameters: 200 μm (27G; Nordson EFD), 250 μm (25G; Nordson EFD) and 330 μm (23G; Nordson EFD). The calibration model 2 was printed in a range of pressures from 12 psi to 20 psi and speed from 5 mm s^−1^ to 25 mm s^−1^ using a 27G tapered nozzle.

### 2.9. Printing Performance Metrics

Printing accuracy was assessed utilizing the measurement of specific distances in printed calibration models using ImageJ (NIH, Bethesda, MD, USA) [33]. Printed models were photographed right after the printing process to prevent drying of the samples and potential deformations.

All of the micro-photographs of samples and additional videos of the printing process were taken using a digital single-lens reflex (DSLR) camera (EOS 700D; Canon, Tokyo, Japan), placed on a firm tripod and under controlled lighting conditions. Images of printed samples’ heights were taken using a USB microscope camera (KKmoon 500×; Digital microscope, Shenzhen, China).

## 3. Results and Discussion

### 3.1. Efficient Calibration for Multi-Material Bioprinting

Bioink P407 was used in the 3D bioprinter calibration, and the evaluation of the printing process. This bioink was selected because of its stable nature, exceptional printability, adequate viscosity, and low swelling [22,34]. Note that the P407 allows for evaluating the capabilities of any bioprinter minimizing the influence of material properties and other secondary factors involved. All of the properties mentioned facilitated the creation of complex architectures and their subsequent evaluation.

**Figure 7:**
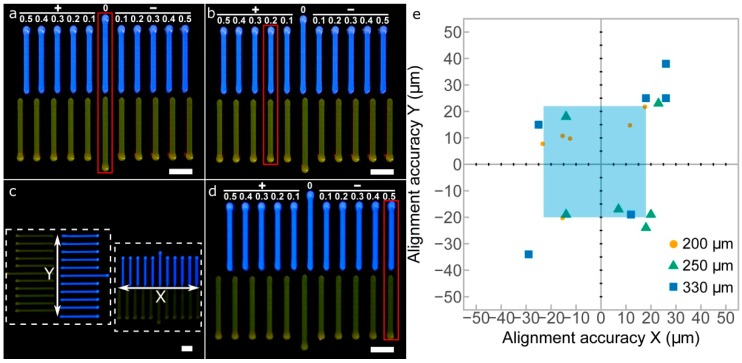
Images of the printed *xy*-offset pattern calibration model between P1 and P2. (**a**) perfect alignment between P1 and P2; (**b**) +200 μm *x*-offset of P2 respect P1; (**c**) overall picture of *x* and *y* calibration models printed at the same time; (**d**) −500 μm *x*-offset of P2 respect P1; (**e**) alignment accuracy in *x* and *y* axes measured for three different nozzle sizes (200 μm, 250 μm, and 330 μm); scale bars: 2 mm.

First, calibration model 1 was printed to perform a quick visual calibration of the *xy* offsets in the four printheads utilized (Figure 7a–d). Calibration errors or deviations in both *x* and *y* axes were measured simultaneously using the printed strand patterns of both axes (Figure 7c). After printing, the patterns allowed the alignment of the printheads P2, P3, and P4 with respect to P1. We considered either positive or negative misalignments in a range between 100 μm and 500 μm. For instance, Figure 7b,d show clear *x*-axis misalignments of +200 μm and −500 μm, respectively. Once the deviations are visually identified, the correction values can be introduced in the slicing software, and the new G-code will correct the position of the printhead nozzles.

The results of the *xy* alignment for three different nozzles are shown in Figure 7e. The increase of the nozzle diameter produced a decrease in the alignment accuracies of both directions. These results can be explained by the much thicker printed lines produced when using bigger nozzles. The same observations, the smaller the nozzle diameter, the higher the line with resolution, were reported by Suntornnond et al. evaluating pluronic F127 [24]. Therefore, it is preferable to perform the 3D printer calibration with the smaller nozzle available. The light blue area indicates the limits for the 200 μm nozzle in the *x* and *y* directions, which obtained the best results of the three nozzles. The maximum alignment errors obtained for this nozzle were in a range from −23 μm to 18 μm in the *x* direction and from −20 μm to 22 μm in the *y* direction. These values are sufficiently low and guarantee that the alignment accuracy is at least of a similar order of magnitude to the mechanical resolution of the 3D printer (20 μm).

**Figure 8:**
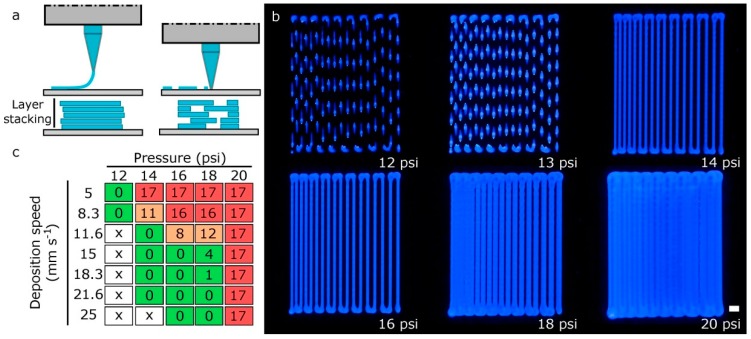
(**a**) scheme of the possible defects in the first layer calibration: nozzle too far from printing platform and nozzle too close to printing platform; (**b**) images of the zigzag path calibration models printed at a deposition speed of 15 mm s^−1^ for various printing pressures; scale bar: 1 mm; (**c**) quantification of the number of filled spaces between strands in the calibration model 2 varying printing pressure and deposition speed (green: good; orange: normal; red: bad; x: discontinuous printing).

The layer-by-layer approach characteristic of additive manufacturing (AM) makes the thickness of the printed layers became the primary factor defining the print resolution along the *z*-axis. When the nozzle is too far from the platform, the printed layers will not adhere to the surface, creating discontinuous strands, and the next layer will not be deposited adequately (Figure 8a). On the other hand, if the tip is too close to the platform, it might lead to a clogging of the nozzle or a discontinuous printing. In some research works, the 3D models are sliced into layers with a slicing height equal to 70% or 80% of the inner needle diameter [26,35]. A lower layer height will result in fewer errors between the layers, but longer printing times. Herein, we found that using a slicing height equal to the nozzle diameter was beneficial when determining the effective deposition rate. Therefore, establishing the right distance between the nozzle and the printing bed for the first layer is of vital importance for avoiding further deposition problems.

The second calibration model or zigzag-path model was useful for determining the printing pressure needed to produce strands of the desired diameter. The variation of the printing pressure in Figure 8b for a fixed deposition speed of 15 mm s^−1^ produced strand widths of different dimensions. As expected, an excessive printing pressure and a low deposition speed produced dramatically wider strands that can eventually overlap (Figure 8c).

### 3.2. Print Resolution in Multi-Material Bioprinting

**Figure 9:**
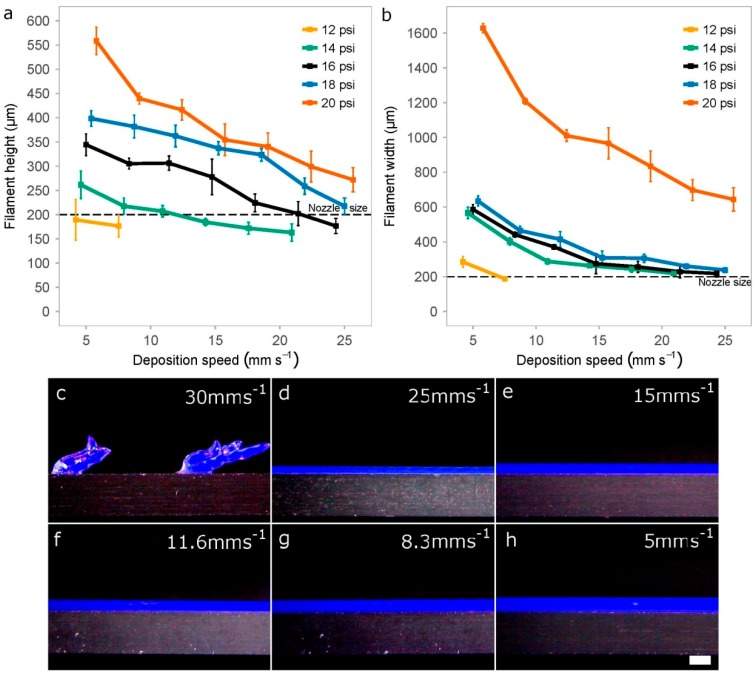
Impact of the printing pressure and deposition speed when creating rectilinear filaments. Quantification of the height (**a**) and width (**b**) of the printed filaments using 40 wt % P407 and a 27G tapered nozzle. Data represents the mean and standard deviation of six different samples (*n* = 6); (**c**–**h**) Representative photographs of different filaments printed at a constant pressure of 16 psi on a cover glass while reducing the deposition speed from 30 mm s^−1^ to 5 mm s^−1^); scale bar: 500 μm.

Extruded hydrogels usually result in spreading or diffusion from the initial shape as a consequence of standing their weight and their slow gelation rates [13]. In addition, the printed strands are never cylindrical, even if we use hollow cylinder-shaped nozzles. For these reasons, we decided to evaluate the print resolution of printed P407 filaments by two dimensions: width and height. We measured these two variables (Figure 9a,b) for different values of printing pressure and deposition speed to identify the optimal printing setup.

We observe that pressure and speed are strongly correlated while working at intermediate pressures (14–20 psi). However, the pressure is probably a more critical factor than deposition speed, especially for the height of the filaments printed (Figure 9a). This is consistent with previous studies on shear thinning hydrogels as the one performed by Trachtenberg et al. printing poly(propylene fumarate) (PPF) [25]. They determined that fiber height and width decreased with increasing deposition speed and decreasing pressure. In addition, they also showed the higher effect of pressure with respect to speed and that the interaction of both factors (pressure and speed) is of great importance.

When printing at very low pressures (12 psi), there was a limitation in the deposition speed (around 8 mm s^−1^) for creating continuous filaments, much lower value than the 25 mm s^−1^ achieved at the pressure of 16 psi. Discontinuous strands were usually generated when printing at higher deposition speed (Figure 9c).

In general, strand width should be almost always greater than height when keeping constant the value of the thickness layer (200 μm) because the nozzle tends to flatten the printed samples. We hypothesized that optimal printing configuration would be that the filaments show similar height and width values with a low swelling ratio. Figure 9a,b demonstrated that these conditions were achieved for printing pressures of 16 psi and 18 psi, and deposition speeds of around 21 mm s^−1^ and 25 mm s^−1^, respectively. Using these parameters and 27G nozzles, the height and width of the strands were very similar: (i) for 16psi was around 202 μm and 230 μm; and (ii) for 18 psi, the height–width values were 219 μm and 238 μm, respectively. Finally, we would like to highlight that very high pressure (20 psi) was a synonym of nonlinear response with too much bioink deposition and diffusion.

### 3.3. Multi-Material Bioprinting of Complex Scaffolds and 3D Constructs

After the four printheads were calibrated in *x*, *y,* and *z* axes and the appropriate setup was found, representative complex structures were printed to demonstrate the goodness of our proposed method. Different bioinks were printed per each layer to study the accumulated misalignments that produce heterogeneous patterns in the lattice scaffolds, and consequently the further reduction of the porosity.

**Figure 10:**
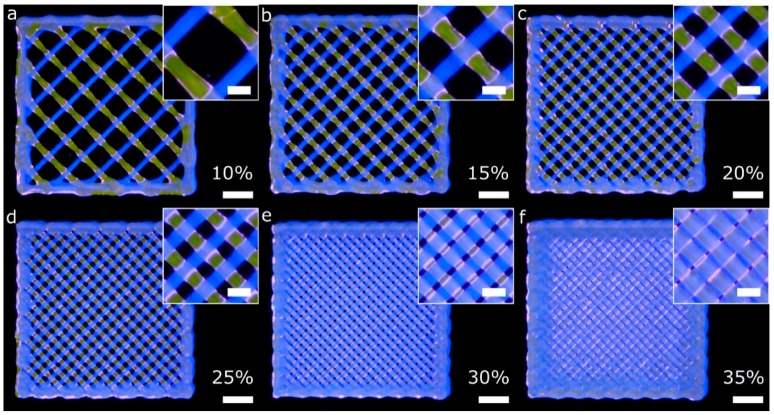
General and detailed views of porous lattice structures printed with two bioinks and two printheads. Each bioink was used in a different layer. The G-code was generated using the slicing software with the infill percentages: 10% (**a**), 15% (**b**), 20% (**c**), 25% (**d**), 30% (**e**) and 35% (**f**). The printing pressure and speed utilized in all the cases were 16 psi and 15 mm s^−1^, respectively; scale bars: 2 mm (general views) and 500 μm (detailed views).

Firstly, porous lattice structures composed of one bioink per layer were printed using two printheads (two fluorescent bioinks). The lattice structures were printed using infill percentages ranging from 10% to 35%. Low and medium infill percentages produced homogeneous patterns across the *xy* plane (Figure 10a–d) because of the successful calibration method. Nevertheless, the higher the infill percentage, the less homogeneous the pattern is. In that case (Figure 10e,f), there was a difference between the theoretical pore area designed and the total pore area printed. The printed pore area was smaller than the theoretical one, similar to what He et al. reported [13].

After printing the first layer, the second layer became a weight load to the first layer at the intersection. In addition, and as explained by [13], the radial diffusion of the upper hydrogel layer on the lower one at the intersections produced a radial narrowing of the pore. As a result, we obtained more rectangular-shaped pores than squared ones. These observations were more evident when the infill density was between 25% and 35% (Figure 10d–f). The limiting higher infill percentage seems to be 30%, with only a few overlapping areas observed. Therefore, we demonstrated that conducting an accurate calibration process is a guarantee of the integrity of the structures created layer-by-layer.

**Figure 11:**
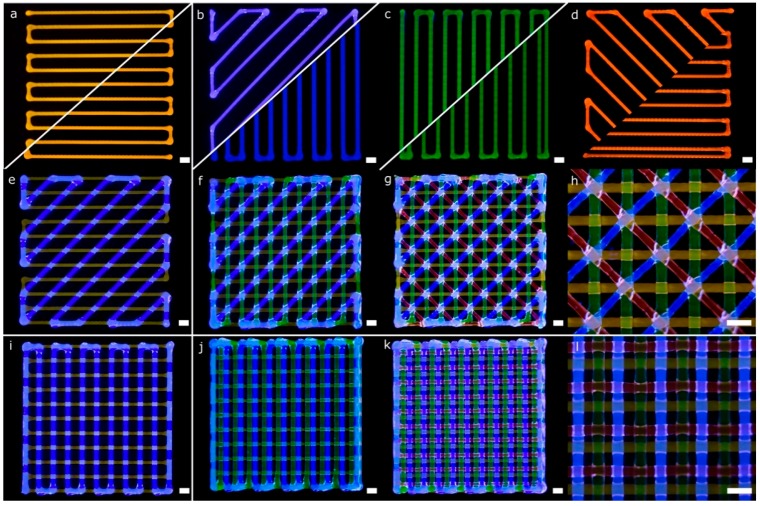
Pictures of a complex porous structures printed using four printheads with parallel and diagonal rectilinear patterns. Each fluorescent bioink was deposited in a different layer (**a**–**d**) with a total of four layers stacked (**e**–**g**,**i**–**k**). Detailed view of the diagonal (**h**) and perpendicular lattice structures (**l**); scale bars: 1 mm.

More complex lattice structures with fluorescent bioinks were printed using the four printheads mounted in the bioprinter. Diagonal and rectilinear patterns (Figure 11a–d) were stacked successfully into two different multi-material scaffolds (Figure 11g,k). The step by step stacking of the layers is depicted in Figure 11e–g,i–k. As in the previous scaffolds, the fidelity at the central part of the structures was better than that at the edges. The lack of accuracy near the edges was due to the accumulation of material in the region where the lines change their angles, similar to the mistakes reported by He et al. for single material extrusion [13]. Looking at the intersection point of the diagonal structure (Figure 11h), we observed that the printheads in charge of dispensing blue and red hydrogels were slightly deviated in the +*x* coordinate (according to Figure 7b). This effect was probably the leading cause of the small dissimilarities in shape observed at the empty triangular areas. These differences were consistent with the geometry tolerances of the structures due to the alignment errors in the *x*-direction reported in the previous sections. In summary, both multi-material structures were printed successfully due to the automatic calibration system used.

Regarding the rectilinear scaffold, the structure was created without overlapping areas (Figure 11i). The diffusion of the upper layer toward the lower one was due to the gravity being more evident than in the previous structure (Figure 11l). This effect is mainly related to the higher infill density (or smaller pore area). Through the successful printing of these two complex scaffolds, the proposed calibration methodology for multi-material bioprinting was verified. We believe that this approach will allow precise control of the deposition of various hydrogels and cell types for the fabrication of more biomimetic tissue structures.

**Figure 12:**
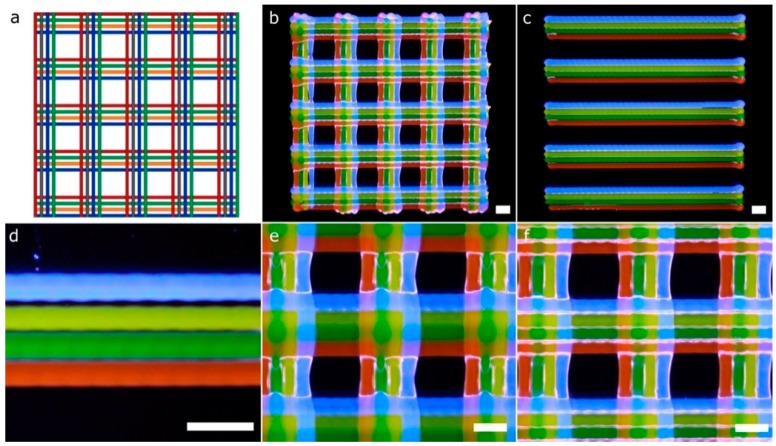
Pictures of a complex porous structure printed using four printheads and rectilinear patterns. The four printheads deposited dyed P407 in the same layer with a total of two layers stacked. General view of the CAD design (**a**) and printed porous structure (**b**); (**c**) general view of the first layer; (**d**) detailed view of printheads misalignments on the *y*-axis; (**e**) detailed view of strands diffusion in the second layer; (**f**) detailed view of the porous structure printed correctly. Scale bars: 1 mm.

Another CAD computer model (Figure 12a), which entails greater complexity compared to the previous structures (Figure 10 and Figure 11), and thereby more calibration requirements, was printed using four fluorescent bioinks (Figure 12b). The model is a lattice structure formed by parallel rectilinear strands, each one with its particular bioink color (Figure 12c). We checked the existence of overlaps or empty spaces between the strands as a sign of an erroneous calibration across the *xy* axes. The overlaps with excessive material accumulated tended to break the continuity of the strands of the next layer (Figure 12e), whereas the errors in the calibration process produced distinct gaps between the parallel strands (see the blue filament in Figure 12d). On the other hand, if the *xy* offsets of the four printheads were correctly determined, the strands were printed without being merged as shown in Figure 12f. Note that the slicing of the 3D models took into account the swelling ratio of the hydrogel P407 (Figure 12a). This ratio was estimated at 100 μm per strand. Therefore, the initial diameter in the computing model needed to be 200 μm to obtain printed strands of 300 μm without overlapping. We conclude that the structures printed are an excellent example of correct calibration cases.

Kang et al. [7] proved the immense potential of these kinds of lattice constructs (Figure 12b) to produce mandible bone and ear-shaped cartilage using cell-laden bioinks side-by-side with PCL to ensure the mechanical strength of the printed constructs. In this paper, we followed a similar approach regarding the MEBB system with four printheads but avoiding the proprietary nature of their multi-material bioprinter. Similar geometries with several bioinks printed right next to the other using parallel rectilinear strands (Figure 12c) but not in a lattice construct were fabricated by Lui et al. [12]. However, their approach incorporates an array of bioink reservoirs routed to a single printhead instead of our multiple and separate printheads. An advantage of the Lui et al. system is that it can eject the bioinks in individually or simultaneously, but it is limited to the use of a single nozzle, which restricts the ability to print hydrogels with very different viscosities. Other multi-material bioprinters such as the 3D-Bioplotter (EnvisionTEC, Gladbeck, Germany) incorporates a mechanism designed to exchange the printheads, which gives flexibility but increases the cost and complexity. Multiple bioinks can be printed in the same 3D model, but increasing the total printing time significantly. Although commercially available 3D bioprinters from EnvisionTEC and RegenHU can assure mechanical resolutions up to 1 µm and 5 µm, respectively, we demonstrated that our system with limited mechanical precision also produced complex structures with enough accuracy for tissue engineering applications [16,25,26,35,36].

**Figure 13:**
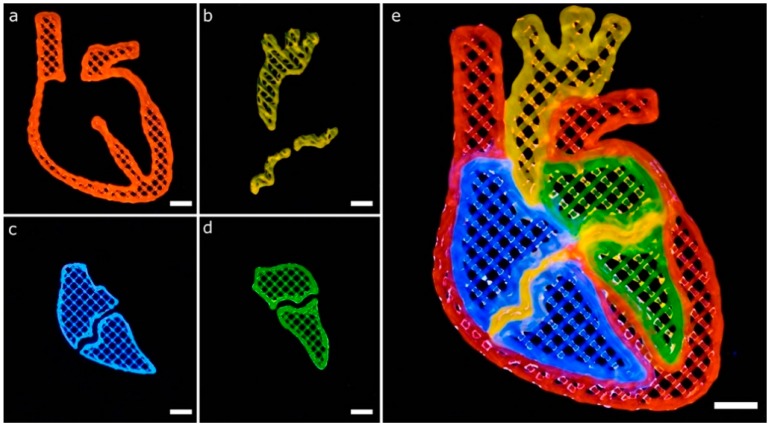
Complex multi-material printing of model that represents a human heart section. The model is composed of heterogeneous bioinks to demonstrate the multi-material capabilities of our system. (**a**–**d**) printing of the main parts of the heart section separately; (**e**) combination of the multiple parts using the four bioinks in a complex structure; scale bars: 5 mm.

Another CAD model to show the potential of a well-calibrated multi-material 3D bioprinter for generating complex structures is depicted in Figure 13. The model represents a human heart section where each of the parts consisted of a single perimeter and a porous infill at 15% printed in two layers. All of the printing trajectories, either for the perimeter or the porous infill (Figure 4b,c), were generated automatically by the slicing software, which greatly facilitated the printing process. We proceed with the following printing sequence: orange (P1), blue (P2), green (P3) and red (P4), but this ordering can be easily changed.

The CAD model of the heart section has curvilinear geometries that create complex trajectories than previous models (Figure 12) based on straight lines. These geometries increased the number of print errors detected. For instance, blue (Figure 13c) and green (Figure 13d) bioinks overlaid the thin middle sections of the heart printed of orange bioink (Figure 13b). Better calibration procedures might avoid these defects by incorporating the effect of the bioink swelling during CAD models generation. Liu et al. printed a very similar geometry of the human heart section using their multi-material platform described before [12]. We obtained similar results with our constructs showing good demarcation among adjacent materials.

**Figure 14:**
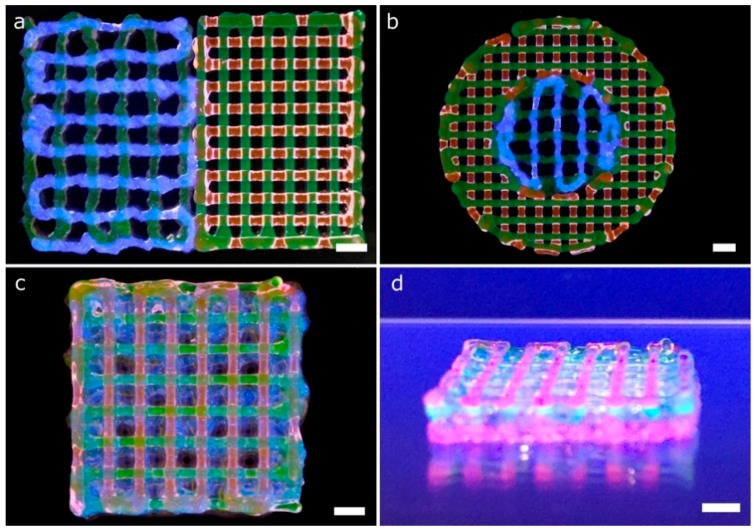
Complex multi-material structures printed. Gel-Alg and P407 bioinks were printed using 25G and 27G tapered nozzles, respectively. (**a**) general view of a 2-layer porous lattice structures printed with Gel-Alg (left, green for printhead 1 and blue for printhead 2) and P407 (right, green for printhead 3 and red for printhead 4) bioinks; (**b**) general view of circular lattice structure with the inner circle printed in Gel-Alg (green for printhead 1 and blue for printhead 2), and the outer circle printed in P407 (green for printhead 3 and red for printhead 4); general (**c**) and side view (**d**) of an 8-layer porous lattice with alternating layers of Gel-Alg and P407. Sequence of colors: Gel-Alg (orange), Gel-Alg (blue), P407 (green) and P407 (red); scale bars: 2 mm.

**Figure 15:**
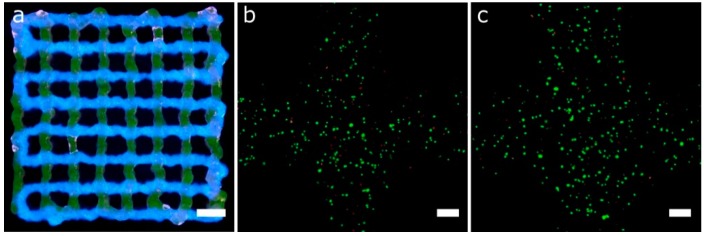
(**a**) general view of 2-layer porous lattice structure printed with Gel-Alg bioinks and two printheads. Each bioink was utilized for a different layer using a 25 G nozzle (scale bar: 2 mm). (**b**,**c**) Representative confocal images of cell viability assay of printed hASCs using the same Gel-Alg mixture and printing parameters than that of (**a**) but without fluorescence inks at 1 h (**b**) and 24 h (**c**) post-printing (scale bars: 200 μm).

Cell-laden Gel-Alg and P407 bioinks were printed in a single session creating multi-material constructs (Figure 14). Gel-Alg represents a more challenging material regarding printability when compared to P407. Consequently, the printed strands were not straight and the openings were irregular, reducing the pore area (Figure 14a). Other authors also reported complications when printing Gel-Alg mixes. Paxton et al. attributed the weak printability to the lower yield stress point of Gel-Alg blends [35]. Despite this, we were capable of calibrating the bioprinter and obtaining the proper printing parameters to create complex constructs from the CAD models in a single multi-material session (Figure 14b–d). High cell viability in the bioprinted Gel-Alg constructs (Figure 15a) was also ensured at 1 h and 24 h post-printing (Figure 15b,c). These observations demonstrated that our calibration method using P407 was helpful in adjusting some printing parameters to specific values that did not reduce cell viability after bioprinting cells with another hydrogel, Gel-Alg blends.

### 3.4. Multi-Material Printing of Complex 3D Vascular Networks

**Figure 16:**
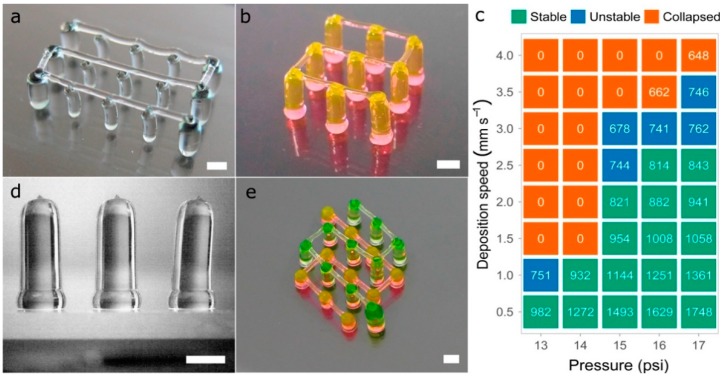
(**a**,**b**) complex vascular 3D networks printed in P407; (**c**) evaluation of the stability of 3D printed vertical pillars for a fixed height of 8 mm. Printing pressure ranging from 13 psi to 17 psi and deposition speeds ranging from 0.5 mm s^−1^ to 4 mm s^−1^. The data inside the figure represents the mean of pillars diameter (in µm) of six different samples (*n* = 6); (**d**) side view of 3D printed 4 mm height pillars; (**e**) vascular structure printed at two different heights (2 mm and 5 mm) with interconnected bridges; scale bars: 1 mm.

Several tests were performed to produce pillars (vertical strands) and hanging bridges between them using P407, similar to the fugitive structures printed by Kolesky et al. that mimic vascular networks [22]. It should be pointed out that the constructs presented in this section were printed using only 40wt % P407 hydrogels colored with different dyes. This does not diminish the widespread of the solution proposed for multi-material bioprinting. Pillars were printed moving the printhead on the *z*-axis and keeping constant the *xy*-coordinates. When printing one pillar, and prior to the printhead movement in the *z*-direction, the tip of the nozzle was placed at 200 μm from the glass slide and the solenoid valve was opened a waiting time of 500 ms. Within this time, the P407 started to flow and permitted to deposit an excess of material in the base of the pillar, in order to give it more stability (Figure 16d). If no waiting time was utilized, a weaker pillar base was produced, decreasing the structure stability. Once the nozzle extruded the pillar moving to the desired height, an additional waiting time of 1 s was considered to allow the column to stabilize. After that, the nozzle was raised at a fast speed (25 mm s^−1^), a distance 2 mm higher than the pillar height to improve its verticality. There was a limit in the column heights that could be achieved without losing verticality.

Different printing pressures (13 psi to 17 psi) and deposition speeds (0.5 mm s^−1^ to 4 mm s^−1^) were tested, producing pillars with different diameters and stability (Figure 16c). A constant pillar height of 8 mm was set for all the vertical pillars printed with a 27G nozzle. As expected, the lower the speed and the higher the pressure, the larger the diameter of the pillars extruded. Regardless of the pressure and the speed utilized, pillars with diameters above 814 μm always remained stable while pillars with diameters between 678 μm and 762 μm tended to bend slightly losing their verticality. Above these diameters, pillars collapsed utterly touching the glass slide.

Regarding the hanging bridges between the pillars, the deposition speed on the *xy*-plane and the distance between pillars have a direct influence on the straightness of the bridge (Figure 16a). To generate continuous straight strands (Figure 16b,e), pillars were spaced up to 4 mm while the deposition speed was set at 7.5 mm s^−1^. When faster deposition speeds were utilized for the bridges, the pillars tended to collapse by the impact of the deposited strands. In general, the samples printed demonstrated excellent results for maximum heights of 8 mm to 10 mm.

If different materials or even the same material but in different concentrations are used to print this type of vascular 3D networks, it would be first necessary to evaluate the stability of the vertical pillars for different process parameters, as shown in this section (Figure 16c). Then, the next step would be to find the optimal deposition speed for the hanging bridges. These types of constructs were tested by Ribeiro et al. [36] printing poloxamer-poly(ethylene glycol) (PEG) blends at different concentrations (poloxamer/PEG: 30%, 29/1%, 28/2%, 27/3%, 26/4% and 20%.). They found that higher concentration of P047 led to a decrease in bridge sagging, which coincides with our observations at higher concentrations of poloxamer.

We agree with He et al. [13] that the ideal multi-material 3D bioprinter for tissue engineering applications should be high throughput, ease of use, with excellent print resolution, and capability of dispensing multiple bioinks with different viscosities. Even if some of the commercially available bioprinters incorporate all these specifications, the authors would like to stand up for the open-source bioprinters. This equipment can provide all discussed advantages plus avoiding the proprietary nature of the commercial ones. Indeed, our open-source 3D printing platform was capable of achieving high accuracy and cell viability in multi-material bioprinting with a relatively lower cost than other commercial units.

### 3.5. Limitations of the Calibration Method in the Multi-Material Bioprinting Proposed

From a mechanical perspective, the calibration of the four printheads depends on the P1 printhead stability, and errors on the P1 are propagated to the other printheads. Similarly, a proper leveling of the printing platform is essential for a successful calibration of the *z*-offset, which becomes even more significant when printing large 3D models. Another limitation of our calibration method is the intrinsic *xy*-offset tolerance that depends on the predefined separation of the printed lines of the pattern proposed called calibration model 1. We assume that our results could dramatically change when printing with very different nozzle diameters or even depending on the material when swelling after printing and the strands’ straightness are not the ideal ones. Moreover, different human errors could cause incorrect choices in the visual identification of the best-aligned pair of strands. Potential users must consider all the tolerances and mistakes mentioned when determining the final print accuracy of their equipment. Concerning the structures printed, critical locations such as sharp corners still need more effort to identify the proper changes in the printing pressure and deposition speed to avoid an excessive accumulation of materials in those areas.

## 4. Conclusions

The use of multiple cell types and biomaterials is essential to recapitulate the architecture, mechanical strength, and complexity of human tissues. In 3D bioprinting, maintaining the print resolution along the layer-by-layer manufacturing process offers greater stability when creating thick self-supporting tissue constructs. We presented a non-expensive and useful calibration method applicable to multi-material 3D bioprinting. The particular multi-material 3D bioprinter herein used was a desktop 3D printer modified to incorporate four independent MEBB printheads.

The base bioink employed for calibration due to its remarkable stability was P407 hydrogel mixed with four fluorescent dyes. Our calibration procedure is exportable to any bioprinting system, but it is strongly recommended to use an automatic *z* offset system to reduce the configuration time drastically. Parameters such as the printing pressure, deposition speed, nozzle height, and nozzle diameter were evaluated from the experimental results to obtain the optimal printing conditions.

Multi-material constructs were printed in different combinations of P407 and Gel-Alg bioinks. In addition, complex multi-material 3D models and intricate vascular networks were created assessing the final accuracy and printing precision of the bioprinting platform. Cell viability after printing cell-laden Gel-Alg bioinks was also verified with successful results. Future works will explore the creation of more complex tissue constructs with different biomaterials and cell types. Other technologies such as drop-on-demand bioprinting could also benefit from the method proposed. Future works could consider the use of different bioprinting technologies to demonstrate the potential and universality of the proposed multi-material calibration method.
